# Analysis and Simulation of the Dynamic Spectrum Allocation Based on Parallel Immune Optimization in Cognitive Wireless Networks

**DOI:** 10.1155/2014/623670

**Published:** 2014-08-28

**Authors:** Wu Huixin, Mo Duo, Li He

**Affiliations:** Department of Information Engineering, North China University of Water Resources and Electric Power, Zhengzhou 450045, China

## Abstract

Spectrum allocation is one of the key issues to improve spectrum efficiency and has become the hot topic in the research of cognitive wireless network. This paper discusses the real-time feature and efficiency of dynamic spectrum allocation and presents a new spectrum allocation algorithm based on the master-slave parallel immune optimization model. The algorithm designs a new encoding scheme for the antibody based on the demand for convergence rate and population diversity. For improving the calculating efficiency, the antibody affinity in the population is calculated in multiple computing nodes at the same time. Simulation results show that the algorithm reduces the total spectrum allocation time and can achieve higher network profits. Compared with traditional serial algorithms, the algorithm proposed in this paper has better speedup ratio and parallel efficiency.

## 1. Introduction

Wireless spectrum is one of the nonrenewable scarce resources. With the rapid development of wireless communication services, wireless spectrum resource is becoming increasingly scarce. The existing research results show that the management system of fixed spectrum has caused huge waste of spectrum resources [1]. In cognitive wireless network, the cognitive users improve the spectrum efficiency effectively by authorizing the “secondary utilization” of spectrum to licensed users (primary users), which is identified as the most effective way at present to solve the contradiction between spectrum resources scarcity and increasing access demands [2, 3].

Spectrum allocation is one of the key issues to improve spectrum efficiency and has become the hot topic in the research of cognitive wireless network. The main goal of spectrum allocation is to assign leisure spectrum resources efficiently to achieve the optimal state under the requirements of cognitive user, network QOS (quality of service), and so on. At present, there are several methods to classify the spectrum allocation. According to different application requirements, the existing spectrum allocation methods mainly include game theory, auction theory, and graph coloring theory [4].

The main idea of spectrum allocation method based on game theory is to map real-time interactive process of cognitive users to game model and to establish a distributed dynamic spectrum sharing mechanism. Neel et al. present various game theory models in cognitive wireless network and analysis of power control, call admission control, and interference avoidance with latent game model [5]. Cao and Zheng propose a bargaining game distributed allocation algorithm. With bargaining game, it is not necessary to recalculate the optimal allocation when the topological structure changes [6]. In [6], the bargaining game trade framework assumes that the network nodes are cooperative; however, network nodes may be selfish in actual system. For the game between noncooperative users, Etkin et al. analyze the efficiency of repeated game using Folk theorem and prove the convergence properties of dynamic spectrum sharing with simulation experiments [7]. Nie and Comaniciu describe formally adaptive channel allocation strategy in cognitive wireless network and, respectively, analyze system performance under the circumstance of cooperative users and noncooperative users [8].

In recent years, auction theory is applied to the spectrum allocation, but the trading way is auction. Successful auction system needs to meet truthfulness, ex post budget balance, and individual rationality [9]. Zhou et al. propose a greedy spectrum allocation algorithm based on computational complexity and truthfulness for the first time and design a bid pricing mechanism based on critical neighbor nodes to ensure the integrity of auction [10]. The algorithm can guarantee integrity of auction, but the allocation result often results in degradation of spectrum utilization. Aiming at this problem, Wang et al. introduce the concept of approximate truthfulness and achieve best tradeoff between the truthfulness of users and the spectrum efficiency [11, 12]. In 2009, Zhou and Zheng apply the concept of double auctions to spectrum auctions and propose new algorithm to ensure that buyers and sellers bid truthfully in double auctions. Inspite of some loss in performance, the algorithm increases the spectrum's using efficiency effectively, and the operation is also predigested much [9].

In this paper we mainly discuss the cooperation based distributed completely limited spectrum allocation algorithm which is implemented based on graph coloring theory. According to Peng's viewpoint, spectrum allocation problem can be equivalent to graph coloring problem, that is, how to select the appropriate channel (color) from channel set of nodes (each channel corresponds to a color) to color each node [13]. In view of the time-varying characteristics of network topology and available channel in cognitive wireless network, Hao et al. put forward two heuristic dynamic spectrum assignment algorithms: fast convergency algorithm with maximum bandwidth (FCMB) and heuristic fairness algorithm with maximum bandwidth (HFWB) [14, 15]. In the existing graph-theory based research, CSGC [13] (color-sensitive graph coloring) algorithm which provides the graph-theory based spectrum allocation model and analyzes the efficiency and fairness in detail is a most representative achievement. The graph-theory based spectrum allocation is an optimization problem and its optimal coloring algorithm is an NP-hard problem. Intelligence optimization is an effective way to solve such problems. In order to reduce the complexity of the algorithm, Zhao et al. introduce genetic algorithm to spectrum allocation and get better allocation results [16]. Later, the artificial-intelligence algorithms such as immune algorithm [17], particle swarm optimization [18, 19], and ant colony algorithm [20, 21] are used to improve the allocation results.

At present, the overall gain of network is a hot point in the existing algorithms, while few related systematic studies in reducing running time of algorithm were reported. In fact, due to the dynamic changes of leisure spectrum information, real-time requirement is one of the notable features to differentiate spectrum allocation in cognitive wireless network from others. Therefore, the time need for spectrum allocation should be as short as possible. Therefore, a spectrum allocation algorithm based on parallel immune optimization is proposed in this paper to reduce the computing time. The experimental results show that the algorithm can achieve higher network profits and can shorten time of spectrum allocation. Compared with traditional serial algorithm, the proposed algorithm can get ideal speedup ratio and efficiency.

## 2. Mathematical Modeling of Spectrum Allocation

To better understand the concept of spectrum allocation, it is necessary to give the mathematical model of spectrum allocation. In this paper we mainly discuss the cooperation based distributed completely limited spectrum allocation algorithm which is implemented based on graph coloring theory. Therefore the spectrum allocation model of cognitive wireless network can be expressed in the following matrices: leisure spectrum matrix *L* (Leisure), benefit matrix *B* (Benefit), constraint matrix *C* (Constraint), and allocation matrix *A* (Allocation).

Suppose that there are *N* cognitive users and *M* leisure spectrums that are all orthogonal to each other. These matrices are stated below.

*(1) Leisure Spectrum Matrix L* *(Leisure)*. Leisure spectrum matrix *L* = {*l*_*n*,*m*_∣*l*_*n*,*m*_∈{0,1}}_*N*×*M*_, where *l*_*n*,*m*_ = 1 denotes that cognitive user *n*  (1 ≤ *n* ≤ *N*) can use spectrum *m*  (1 ≤ *m* ≤ *M*) and *l*_*n*,*m*_ = 0 denotes that cognitive user *n* cannot use spectrum *m*.

*(2) Benefit Matrix B* *(Benefit)*. In the same leisure spectrum, the benefit achieved by different cognitive users can be represented by benefit matrix *B* = {*b*_*n*,*m*_}_*N*×*M*_, that is, the benefit user *n*  (1 ≤ *n* ≤ *N*) gains from using spectrum *m*  (1 ≤ *m* ≤ *M*). Obviously, when *l*_*n*,*m*_ = 0, there will be *b*_*n*,*m*_ = 0; that is to say, only the leisure spectrum has benefit matrix.

*(3) Constraint Matrix C* *(Constraint)*. Different cognitive users can sometimes share the same leisure spectrum. This means that more spectrum resources are available, but it may cause interference which can be represented by constraint matrix *C* = {*c*_*n*,*k*,*m*_∣*c*_*n*,*k*,*m*_∈{0,1}}_*N*×*N*×*M*_, where *c*_*n*,*k*,*m*_ = 1 denotes that the interference will be caused when cognitive users *n* and *k*  (1 ≤ *n*, *k* ≤ *N*) simultaneously use spectrum *m*  (1 ≤ *m* ≤ *M*); otherwise, *c*_*n*,*k*,*m*_ = 0. The constraint matrix *C* is determined by the leisure spectrum matrix. When *n* = *k*, we can get that *c*_*n*,*n*,*m*_ = 1 − *l*_*n*,*m*_ and *c*_*n*,*k*,*m*_ ≤ *l*_*n*,*m*_ × *l*_*k*,*m*_. That is to say, only spectrum *m* is available to cognitive users *n* and *k* simultaneously; the interference may be caused.

*(4) Noninterference Allocation Matrix A*. Assign the leisure and noninterference spectrum to cognitive users. We can get noninterference allocation matrix: *A* = {*a*_*n*,*m*_∣*a*_*n*,*m*_∈{0,1}}_*N*×*M*_, where *A* is equal to zero or 1 and *a*_*n*,*m*_ = 1 denotes assigning the spectrum *m* to cognitive user *n*. Otherwise, *a*_*n*,*m*_ = 0. Allocation matrix must satisfy the following constraints defined by *C*:

(1) an,m×ak,m=0, if  cn,k,m=1,  ∀n,k<N,  m<M.

From the above description we can see that more than one allocation matrix satisfies the conditions. Let Λ*N*, *M* be the set of all allocation matrices. For a given noninterference allocation matrix *A*, the total revenue earned by secondary user *n* can be represented by benefit vector *R*:

(2) $$\begin{array}{c} R={\left\{r_{n}=\overset{M}{\underset{m=1}{\sum }}a_{n,m}\times b_{n,m}\right\}}_{N\times 1}. \end{array}$$

The primary goal of spectrum allocation is to maximize the network efficiency *U*(*R*), which can be represented as the following optimization problem:

(3) $$\begin{array}{c} A^{*}=\underset{A\in \wedge \left(L,C\right)N,M}{\text{arg max}}U\left(R\right), \end{array}$$

where arg(·) denotes the corresponding spectrum allocation matrix when maximizing the network efficiency. Therefore, A* is the most optimal noninterference spectrum allocation matrix.

It is clear that *U*(*R*) has different representations [4, 17]. Considering the demand for network flow and fairness, this paper defines *U*(*R*) with the following three forms [17, 22].

*(1) The Sum of the Maximized Network Efficiency (MSR)*. The goal is to maximize the system total revenue and the optimization problem can be expressed as follows:

(4) $$\begin{array}{c} U_{\text{sum}}=\overset{N}{\underset{n=1}{\sum }}r_{n}=\overset{N}{\underset{n=1}{\sum }}\;\overset{M}{\underset{m=1}{\sum }}a_{n,m}\times b_{n,m}. \end{array}$$

In order to adopt the same evaluation standard with the following two revenue functions, this paper uses average revenue instead of total revenue. The sum of average maximized network revenue (MSRM) is defined as follows:

(5) $$\begin{array}{c} U_{\text{mean}}=\frac{1}{N}\overset{N}{\underset{n=1}{\sum }}r_{n}=\frac{1}{N}\overset{N}{\underset{n=1}{\sum }}\;\overset{M}{\underset{m=1}{\sum }}a_{n,m}\times b_{n,m}. \end{array}$$

*(2) Maximized Minimum Bandwidth (MMR)*. The goal is to maximize the spectrum efficiency of restricted user (bottleneck user) and the optimization problem can be expressed as follows:

(6) $$\begin{array}{c} U_{\min}=\underset{1\leq n\leq N}{\min}r_{n}=\underset{1\leq n\leq N}{\min}\left(\overset{M}{\underset{m=1}{\sum }}a_{n,m}\times b_{n,m}\right). \end{array}$$

*(3) Maximized Proportion Fairness (MPF)*. The goal is to consider the fairness of each user. To ensure that *U*_mean_ and *U*_min⁡_ are comparable, the fairness measure can be defined as follows:

(7) $$\begin{array}{c} U_{\text{fair}}={\left(\overset{N}{\underset{n=1}{\prod }}r_{n}\right)}^{1/N}={\left(\overset{N}{\underset{n=1}{\prod }}\;\overset{M}{\underset{m=1}{\sum }}a_{n,m}\times b_{n,m}+10^{-4}\right)}^{1/N}. \end{array}$$

Therefore, under the same allocation, *U*_mean_ ≥ *U*_fair_ ≥ *U*_min⁡_.

## 3. Implementation of Spectrum Allocation Based on Parallel Immune Optimization

### 3.1. Main Idea of the Algorithm

It can be seen from the above analysis that the main problem of spectrum allocation is how to find the most optimal spectrum allocation matrix *A* so as to maximize network benefit *U*(*R*) when *L*, *B*, and *C* are known.

In order to accelerate the convergence rate of intelligent optimization algorithms and also keep the population diversity, parallel implementation is an effective way. Using the evolutionary algorithms for reference, parallelization models of artificial immune optimization mainly include master-slave parallel model, coarse-grained parallel model, and fine-grained parallel model [23–25]. In this paper, master-slave parallel model is adopted for its simpleness and tending to realize. It is a single population evolution model, divided into master process and slave process [26, 27]. The master process executes evolution operator while the slave process computes the affinity of each antibody, respectively. And they work alternatively.

Antibody is mapped to the candidate solution to the problem in artificial immune optimization algorithm. The algorithm will obtain the required solution eventually by optimizing the antibody through initialization, affinity evaluation, clonal expansion, clonal variation, and clonal selection [28, 29]. For artificial immune optimization algorithm, the traditional serial algorithm circularly computes the affinity of each individual after cloning the individual, while in parallel computing, the circulation can be decomposed [21, 30]. That is to say, the individuals are sent to many nodes in the cluster through message; and then their affinity are computed parallel; finally each node sends the computed affinity back.

Parallel algorithm process is described simply as follows.

1. The master node obtains the number of CPUs (*X*) and population size (*Y*).
2. For each node, calculate the number of individuals (*Z*). The number of individuals for each CPU to calculate is *Z* = ⌊*X*/*Y*⌋ (rounding down) and the rest of the individuals are sent to the higher-numbered CPU. Therefore, the number of individuals each CPU receives in this approach is not more than one at most.
3. The master nodes send individual coding to slave nodes; then each slave node computes the affinity parallel and sends back the result to master node. The master node receives the affinity value from slave node.

### 3.2. Explanation of the Key Techniques

For spectrum allocation optimization problem, the key technologies of immune optimization algorithm designed in this paper are described as follows.

*(1) Antibody Encoding*. Encoding is the process of mapping the problems to be solved to immune antibody. According to the characteristics of the problems, the simplest way to obtain the allocation matrix *A* is to use matrix encoding. But due to the fact that many elements of *L* are zero, this causes many elements of the corresponding allocation matrix to be zero too [31–33]. Hence, it will waste considerable storage space to use matrix storage. In this paper, only the elements of matrix *A* that correspond to the elements whose value is one in *L* are encoded. So the number of such elements in *L* is *l* = ∑_*n*=1_^*N*^∑_*m*=1_^*M*^ *l*_*n*,*m*_. Each antibody represents a possible spectrum allocation scheme and this encoding method reduces the search space effectively.

*(2) Mapping from Antibody Encoding to Allocation Matrix A*. In matrix *L*, let *n* and *m* denote the subscripts of the elements whose value is equal to 1 and be kept in *L*_1_ in incremental way first according to *n* and then *m*; that is, *L*_1_ = {(*n*, *m*)∣*l*_*n*,*m*_ = 1}. Obviously, the number of elements in *L*_1_ is *l*. Then each digit of each antibody *j*  (1 ≤ *j* ≤ *l*) is mapped to *a*_*n*,*m*_, that is, the elements of matrix *A*, where the value of (*n*, *m*) is *j*  (1 ≤ *j* ≤ *l*).

*(3) Representation of Affinity Function*. The goal of spectrum allocation is to maximize the network benefit *U*(*R*), so we use *U*(*R*) as affinity function directly.

### 3.3. Implementation Steps of the Algorithm

In this paper, the implementation steps of the algorithm are as follows: (where *P* denotes antibody population and *P*_*i*_ denotes an antibody).

**Step 1 (initialization).**

Let evolutional generation *g* = 0 and let population size be *s* (size). According to the encoding pattern discussed above, random initialization population *P*(*g*) = {*P*_1_(*g*), *P*_2_(*g*) ⋯ *P*_*s*_(*g*)}.

**Step 2 (mapping from antibody representation to allocation scheme).**

With the antibody mapping pattern, each antibody is mapped to corresponding allocation matrix *A*, that is, a possible spectrum allocation scheme.

**Step 3 (processing the interference constraint).**

The allocation matrix *A* produced in Step 2 meets the requirement of *C* uncertainly, so it should be revised. The implementation procedure is as follows: for any *m*, if *c*_*n*,*k*,*m*_ = 1, then check whether the elements in line *n* and *k* of the column *m* are equal to 1 simultaneously. If so, set one of the two elements to zero at random and keep the other unchanged. In this way, the allocation matrix *A* obtained is a feasible solution. Meanwhile, *P*(*g*) should be updated by mapping the corresponding antibody.

**Step 4 (according to the above parallelization process, evaluating *P*(*g*) with affinity function).**

Compute the affinity of the *s* antibodies in *P*(*g*) and sort them in descending order. Obviously, the corresponding allocation matrix *A* of the antibody whose affinity value is the biggest of all is namely the optimal allocation scheme.

**Step 5 (checking termination conditions).**

The algorithm will terminate if the number of iterations reaches the set threshold (*g*_max⁡_). Map the antibody with the highest affinity value in the antibody population to *A*, that is, the optimal spectrum allocation (the focus of this article). Otherwise, go to Step 6.

**Step 6 (clonal expansion *T*_*g*_^*C*^).**

The antibodies in *P*(*g*) are cloned in adaptive mode, that is, the higher the antibody affinity and the smaller the antibody concentration is, the larger the clone scale will be, which is beneficial to keep population diversity and avoid premature convergence. After the clone operation, the antibody population is marked as *P*′(*g*).

**Step 7 (clonal variation *T*_*g*_^*m*^).**

Based on the probability *p*_*m*_, the simple mutation [34, 35] operation *T*_*g*_^*C*^ is carried out in population *P*′(*g*); then we will obtain the antibody population *P*′′(*g*).

**Step 8 (clonal selection *T*_*s*_^*c*^).**

If the population size is less than *s* after clone operation, new antibodies will be generated randomly to replenish it. Otherwise, select the top *s* antibodies to form new antibody population *P*(*g* + 1) = *T*_*s*_^*c*^(*P*′′(*g*)). Then go to Step 2.

## 4. Simulation Experiment and Result Analysis

### 4.1. Simulation Environment and Parameter Settings

The algorithm is realized on HPC cluster in parallel computing lab. There is a management node, an I/O node, and 32 conventional computing nodes in the HPC cluster. Operating system is RedHat Enterprise Linux AS and C+ MPI is used for programming implementation. MPI is the international standards of message-passing model. In the course of the experiment, the matrices *L*, *B*, and *C* are generated by the pseudocode listed in the literature [13] and they are ensured to meet the corresponding constraint. In this paper, the parameters in immune algorithm are set as follows: maximum evolutional generation *g*_max⁡_ = 200, population size = 20, mutation probability *p*_*m*_ = 0.1, and controlling parameters for clone scale *n*_*c*_ = 5 and the number of CPUs is 1 to 4.

### 4.2. Experimental Results and Analysis

In order to validate the performance of this algorithm, we compare it with the classic spectrum allocation algorithm CSGC [13], immune clonal selection algorithm [17], and GA-SA (GA-spectrum allocation) [36]. These three algorithms which work on I/O nodes are serial algorithms. Experimental result is measured with the network benefits. For fair comparison, we use the same matrices *L*, *B*, and *C* and the same parameter settings and run the algorithm 50 times to take the average result. The average benefits of 50 times experiments are listed in Tables 1 and 2, where *M* = *N* = 5 and *M* = *N* = 20, respectively (*M* and *N* denote the leisure spectrum and cognitive user, resp.).

From Tables 1 and 2, we can see that the network benefits including MSRM, MMR, and MPF in this paper are all higher than the other three typical algorithms. Meanwhile, with the increase of iteration times (CSGC is deterministic algorithm and does not change with the iteration times), convergence speed of this algorithm is faster than that of serial immune algorithms, which shows that this algorithm has satisfactory result with fast solution speed.

**Figure 1:**
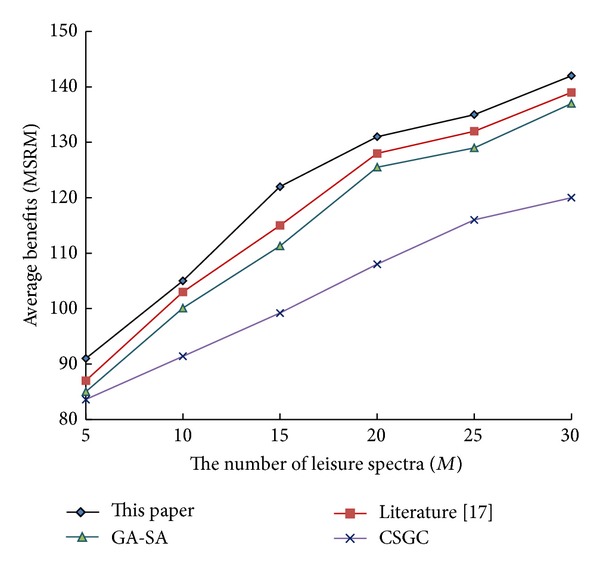
Influence of leisure spectrum on MSRM.

**Figure 2:**
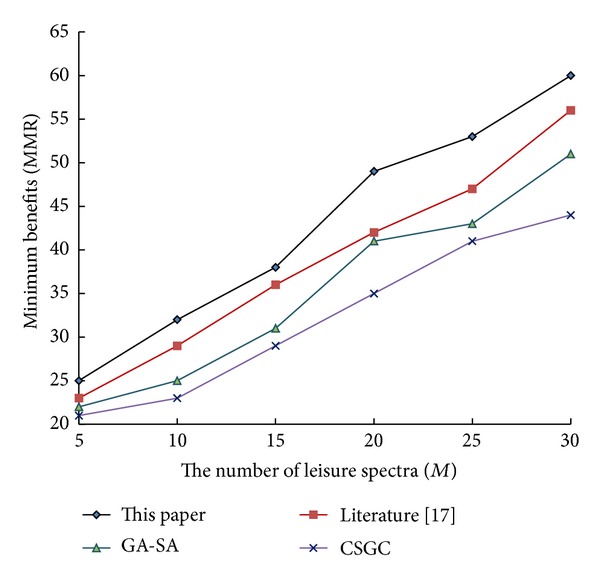
Influence of leisure spectrum on MMR.

**Figure 3:**
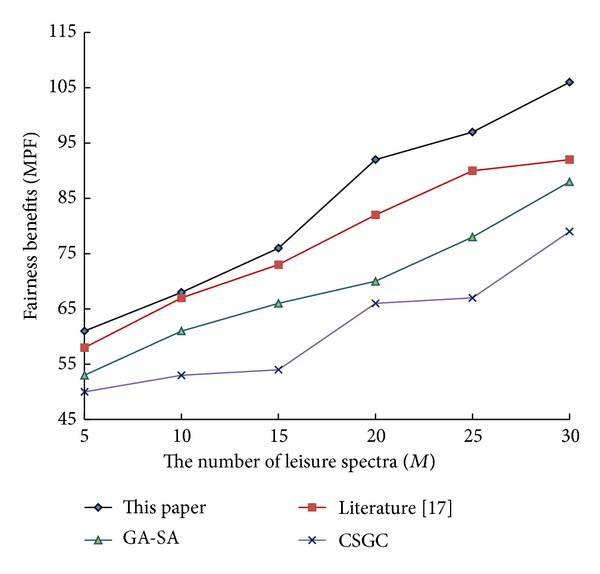
Influence of leisure spectrum on MPF.

To further validate the algorithm, let cognitive users *N* remain unchanged (*N* = 10). The performance of the relevant algorithms is shown in Figures 1, 2, and 3 with the increase of leisure spectrum *M*.

**Figure 4:**
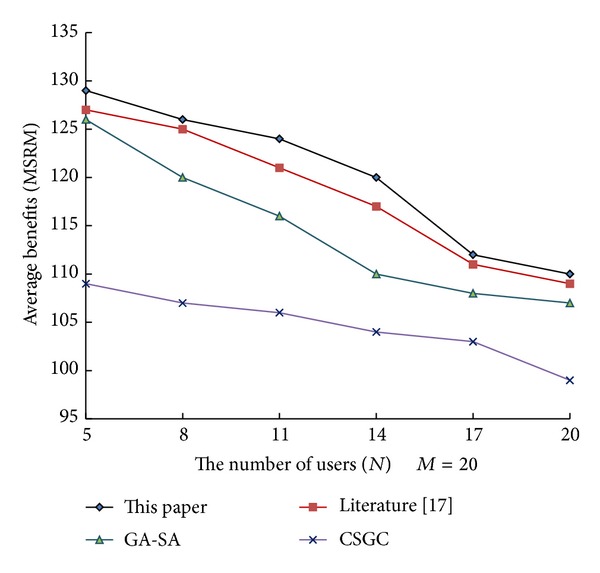
Influence of number of cognitive users on MSRM.

**Figure 5:**
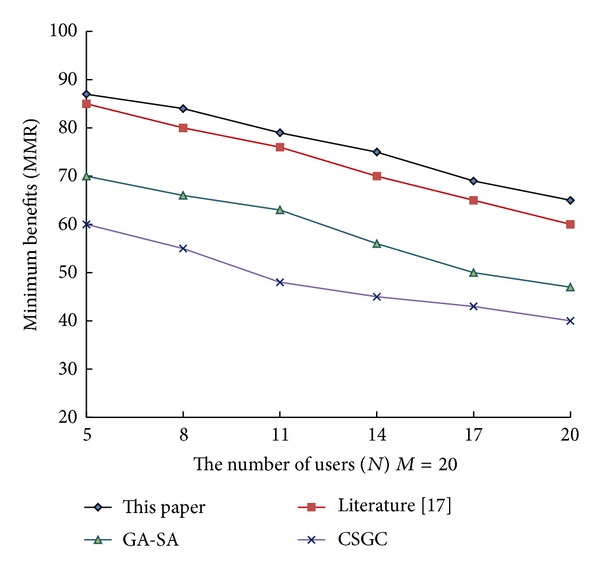
Influence of number of cognitive users on MMR.

**Figure 6:**
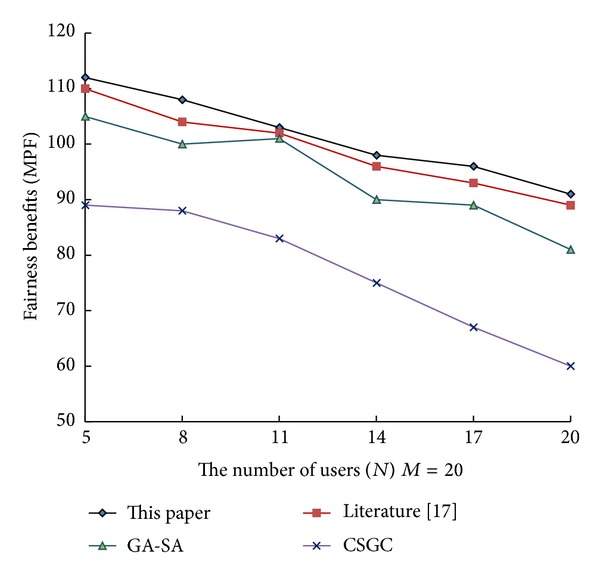
Influence of number of cognitive users on MPF.

From Figure 1 to Figure 3, it can be see that network benefits increase with the increase of leisure spectrum *M*. Experimental results show that this algorithm is superior to the three existing classical algorithms in terms of network benefits (MSRM, MMR, and MPF), which further indicates the effectiveness of the algorithm. Meanwhile, the experiments also verity the influence of the number of cognitive users on network benefits in the case that the leisure spectrum *M* is known (*M* = 20), which is shown in Figures 4, 5, and 6. The experimental results show that with the increase of cognitive users, system benefits will decrease. But the benefit of this algorithm is superior to the other three existing algorithms, which proves its superiority.

In addition, the theoretically optimal allocation can be viewed as upper limit of network performance. The theoretically optimal allocation can be obtained by exhaustive search. However, the optimal algorithm of spectrum allocation is an NP-hard problem; solution space increases exponentially with the problem scale magnifying. In order to ensure the computational complexity of exhaustive search is feasible, we let *M* = *N* = 5 in the following contrast experiment [17]. The calculation method of relative error is as follows: if optimal value of network benefits obtained by one algorithm is *T* and the theoretically optimal is *T*_opt_, then the relative error is 1 − *T*/*T*_opt_.  Table 3 lists the relative error of different algorithm.

From Table 3 we can see that the relative error of the algorithm proposed in this paper is smaller than the others. After about 100 iterations with this algorithm, the results are very close to the optimal solution. And after 200 iterations, the optimal solution can be obtained basically which proves its superiority from another side.

### 4.3. Performance Analysis of Parallel Algorithm

In general, speedup ratio and efficiency analyses [23–25] are used to evaluate the relative benefits of parallel algorithms. Speedup ratio *s*_*p*_ = *t*_*s*_/*t*_*p*_, where *t*_*s*_ is the running time of serial algorithm to solve a problem and *t*_*p*_ is the running time of parallel algorithm to solve the same problem. Thus it can be seen that speedup ratio is the degree that the parallelism of algorithm is expected to improve the running time. Efficiency *E*_*p*_ = *s*_*p*_/*p*, where *p* is the number of CPUs. Efficiency reflects the effective utilization of the processor in parallel system. Here, we compare the algorithm with serial immune clone algorithm discussed in the literature [17] under the scale of problem *M* = *N* = 20.

Table 4 shows the running time, speedup ratio, and efficiency of spectrum allocation algorithm with different number of CPUs.

As can be seen from Table 4, with the increase of the number of CPUs, the running time of algorithm will decrease by not more than 50%. The major reasons are as follows.

1. System computational power induces more significant effect on parallel algorithm than serial algorithm. With the increase of the number of CPUs, the running time of parallel algorithm decreases rapidly; however, the downtrend of serial algorithm is gentler.
2. The part of parallel program is sample, which makes the percentage of the execution of serial program portion increase.
3. There are some limiting factors in hardware and network settings. In addition, with the increase of the CPU number, speedup ratio increases obviously but the efficiency reduces. The main reason is that the amount of computation for each CPU decreases as the number of CPUs increases. In this way the proportion of data transmission time to the whole time will increase, which results in decrease of efficiency gradually.

## 5. Conclusion and Ongoing Work

Spectrum allocation is one of the key issues in cognitive wireless network, and real-time performance is one of its notable characteristics. In this paper, we define a general mathematical model of spectrum allocation. By reducing the optimal allocation to one of color-sensitive graph coloring (CSGC), we show that it is an NP-hard problem. While taking into account spectrum heterogeneity, we propose a spectrum allocation algorithm based on parallel immune optimization and give the detailed steps of the implementation. Meanwhile, we set up a set of evaluation indices of algorithm performance. Our experimental results show that our algorithm not only can drastically improve network benefits but also shortens the time of spectrum allocation and promotes the real-time performance of cognitive spectrum allocation.

In the simulation, we assume that the available spectra are static during the time they takes to perform spectrum assignment. But if a dynamic network is considered, spectrum allocation becomes a more complex problem and the algorithm needs to recompute allocations as the topology changes. So, an adaptive approach should be developed to adapt the environment change and the change of spectrum availability. Meanwhile, the load balancing and program optimization of the dynamic network, the parallel model of parallel immune optimization algorithm, and the relation between the subpopulation size and computing power also need further research.

Furthermore, the researches of this paper are mainly oriented toward qualitative analysis; therefore it is necessary to do some quantitative analysis in the next step to verify its superiority in the actual communication environment.

## Figures and Tables

**Table 1:** Network benefits comparison with other classic serial algorithms under different iteration times (*M* = *N* = 5).

Iteration times	Algorithms	MSRM	MMR	MPF
20	This paper	82.05	23.42	59.91
	GA-SA	76.37	20.58	52.46
	Literature [17]	81.68	21.98	57.23

100	This paper	89.88	24.31	60.83
	GA-SA	88.42	21.60	53.98
	Literature [17]	89.50	23.20	58.26

200	This paper	89.88	24.37	60.89
	GA-SA	88.48	22.54	54.23
	Literature [17]	89.50	23.20	58.26

	CSGC	83.26	20.27	50.02

**Table 2:** Network benefits comparison with other algorithms (*M* = *N* = 20).

Iteration times	Algorithms	MSRM	MMR	MPF
20	This paper	105.26	31.23	69.79
	GA-SA	100.37	27.56	52.38
	Literature [17]	104.26	29.68	67.65

100	This paper	109.26	37.95	92.01
	GA-SA	100.82	32.68	76.34
	Literature [17]	108.54	36.26	88.23

200	This paper	109.73	55.41	92.95
	GA-SA	106.82	42.54	78.65
	Literature [17]	108.54	53.25	88.47

	CSGC	98.74	36.23	60.12

**Table 3:** Relative error of different algorithms under different iteration times (*M* = *N* = 5).

Iteration times	Algorithms	Relative error (%)		
		MSRM	MMR	MPF
20	This paper	0.041	0.501	2.106
	GA-SA	0.372	3.569	3.389
	Literature [17]	0.056	0.582	2.650

100	This paper	0.005	0.291	1.628
	GA-SA	0.058	2.682	2.342
	Literature [17]	0.006	0.328	1.832

200	This paper	0	0	1.001
	GA-SA	0.054	2.544	3.650
	Literature [17]	0	0	1.275

	CSGC	0.622	3.238	6.124

**Table 4:** Performance of parallel algorithm in running time, speedup ratio, and efficiency with the different number of CPUs (*M* = *N* = 20).

The number of CPUs (*P*)	Algorithms	Running time (s)	Speedup ratio (*t*_*s*_/*t*_*p*_)	Efficiency (*s*_*p*_/*p*)
1	This paper Literature [17]	182.4 242.3	1.34	1.34

2	This paper Literature [17]	116.7 201.9	1.73	0.865

3	This paper Literature [17]	61.5 146.7	2.39	0.797

4	This paper Literature [17]	40.9 119.1	2.91	0.728
